# Lynch syndrome for the gynaecologist

**DOI:** 10.1111/tog.12706

**Published:** 2021-01-18

**Authors:** Neil AJ Ryan, Raymond FT McMahon, Neal C Ramchander, Mourad W Seif, D Gareth Evans, Emma J Crosbie

**Affiliations:** ^1^ Obstetrics and Gynaecology Specialty Registrar and Honorary Clinical Lecturer Centre for Academic Women’s Health University of Bristol Bristol UK; ^2^ Consultant Histopathologist and Emeritus Professor of Medical Education Department of Histopathology Manchester University NHS Foundation Trust Manchester Academic Health Science Centre Manchester UK; ^3^ Foundation Programme Doctor Division of Cancer Sciences Faculty of Biology, Medicine and Health University of Manchester St Mary's Hospital Manchester UK; ^4^ Consultant Gynaecologist and Honorary Senior Lecturer Division of Gynaecology St Mary’s Hospital Manchester University NHS Foundation Trust Manchester Academic Health Science Centre Manchester UK; ^5^ Professor of Medical Genetics and Cancer Epidemiology and Honorary Consultant in Medical Genetics Division of Evolution and Genomic Medicine University of Manchester St Mary's Hospital Manchester UK; ^6^ Professor of Gynaecology Oncology and Honorary Consultant Gynaecological Oncologist Division of Cancer Sciences Faculty of Biology, Medicine and Health University of Manchester St Mary's Hospital Manchester UK

**Keywords:** endometrial cancer, genetic predisposition, Lynch syndrome, mismatch repair, ovarian cancer

## Abstract

**Key content:**

- Lynch syndrome is an autosomal dominant condition closely associated with colorectal, endometrial and ovarian cancer.
- Women with Lynch syndrome are at increased risk of both endometrial and ovarian cancer and should be offered personalised counselling regarding family planning, red flag symptoms and risk‐reducing strategies.
- Surveillance for gynaecological cancer in women with Lynch syndrome remains controversial; more robust data are needed to determine its effectiveness.
- Universal testing for Lynch syndrome in endometrial cancer is being adopted by centres across Europe and is now recommended by the National Institute for Health and Care Excellence; thus, gynaecologists must become familiar with testing strategies and their results.
- Testing strategies involve risk stratification of cancers based on phenotypical features and definitive germline testing.

**Learning objectives:**

- To define the pathogenesis of Lynch syndrome and its associated gynaecological cancers.
- To understand the testing strategies for Lynch syndrome in women with gynaecological cancer.
- To learn how best to counsel women with Lynch syndrome regarding gynaecological cancer and risk‐reducing strategies to enable informed decision‐making.

**Ethical issues:**

- Offering gynaecological surveillance despite a lack of robust evidence for its clinical effectiveness may falsely reassure women and delay risk‐reducing hysterectomy.
- Genetic testing may yield variants of unknown significance with ill‐defined clinical implications, which can lead to confusion and anxiety.
- Genetic testing has implications not only for the individual, but also for the whole family, so expert counselling is crucial.

## Introduction

Genetics has become an integral part of our specialty, informing prenatal diagnosis, fertility investigations, the management of gynaecological cancers and many other aspects of women’s health care. Genomics England has now completed its sequencing of 100 000 genomes and has established a workable infrastructure for continuing gene and genome sequencing within the UK’s National Health Service (NHS). Soon, clinicians will have access to a national genomic test directory^1^ and will be encouraged to order genetic testing for their patients. In parallel, ever‐increasing numbers of people are taking private genetic tests and looking to their doctors to explain the results. With the integration of genomic medicine into routine clinical practice, obstetricians and gynaecologists must become familiar with common genetic conditions. One such condition is Lynch syndrome.

Lynch syndrome is an autosomal dominant inherited condition that predisposes an individual to a constellation of cancers, including colorectal, endometrial and ovarian cancer. It is thought to be the most common high penetrance inherited predisposition to cancer, with most affected people unaware of their risk.^2^ Gynaecological cancer is often the first cancer diagnosis in women with Lynch syndrome.^3^ This provides an opportunity to diagnose Lynch syndrome before they or their family are affected by further oncological sequelae. Early diagnosis allows women to be enrolled in cancer surveillance programmes and enables cascade testing for their at‐risk family members. There is a well‐documented survival advantage for those with Lynch syndrome who are compliant with colonoscopic surveillance for bowel polyps.^4^ In addition, early identification of Lynch syndrome can enable the uptake of cancer risk‐reducing strategies, including taking aspirin and lifestyle modification. The gynaecologist, therefore, has a crucial role in diagnosing Lynch syndrome and advising women of its implications.

## Lynch syndrome

**Figure 1:**
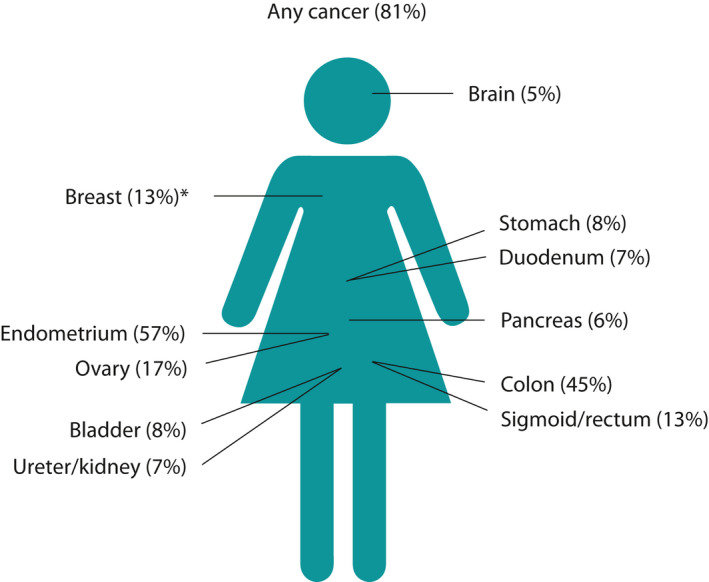
Percentage maximum risk of cancer in females at 75 years of age across different pathogenic gene variants. *In path_PMS2, the risk of breast cancer could be as high as 55%, but the data are of poor quality because of low incidence.

Lynch syndrome was first described by Aldred Warthin in 1913 and was further delineated by Henry Lynch in 1966, after whom the condition is named.^5^ In these seminal pedigrees, it was endometrial cancer that predominated. The cancers associated with Lynch syndrome are shown in Figure 1.

Lynch syndrome arises from inherited mutations, known as pathogenic variants, in the genes encoding the proteins of the highly conserved DNA mismatch repair (MMR) system: mutL homolog 1 (*MLH1*), mutS homolog 2 (*MSH2*), mutS homolog 6 (*MSH6*) and PMS1 homolog 2 (*PMS2*).^6^ Deletions involving epithelial cell adhesion molecule (*EpCAM*) can lead to downstream epigenetic silencing of *MSH2*.^7^ Less commonly, inherited inactivation of the MMR system can arise from germline hypermethylation of the promoter region of *MLH1*.^8^

**Figure 2:**
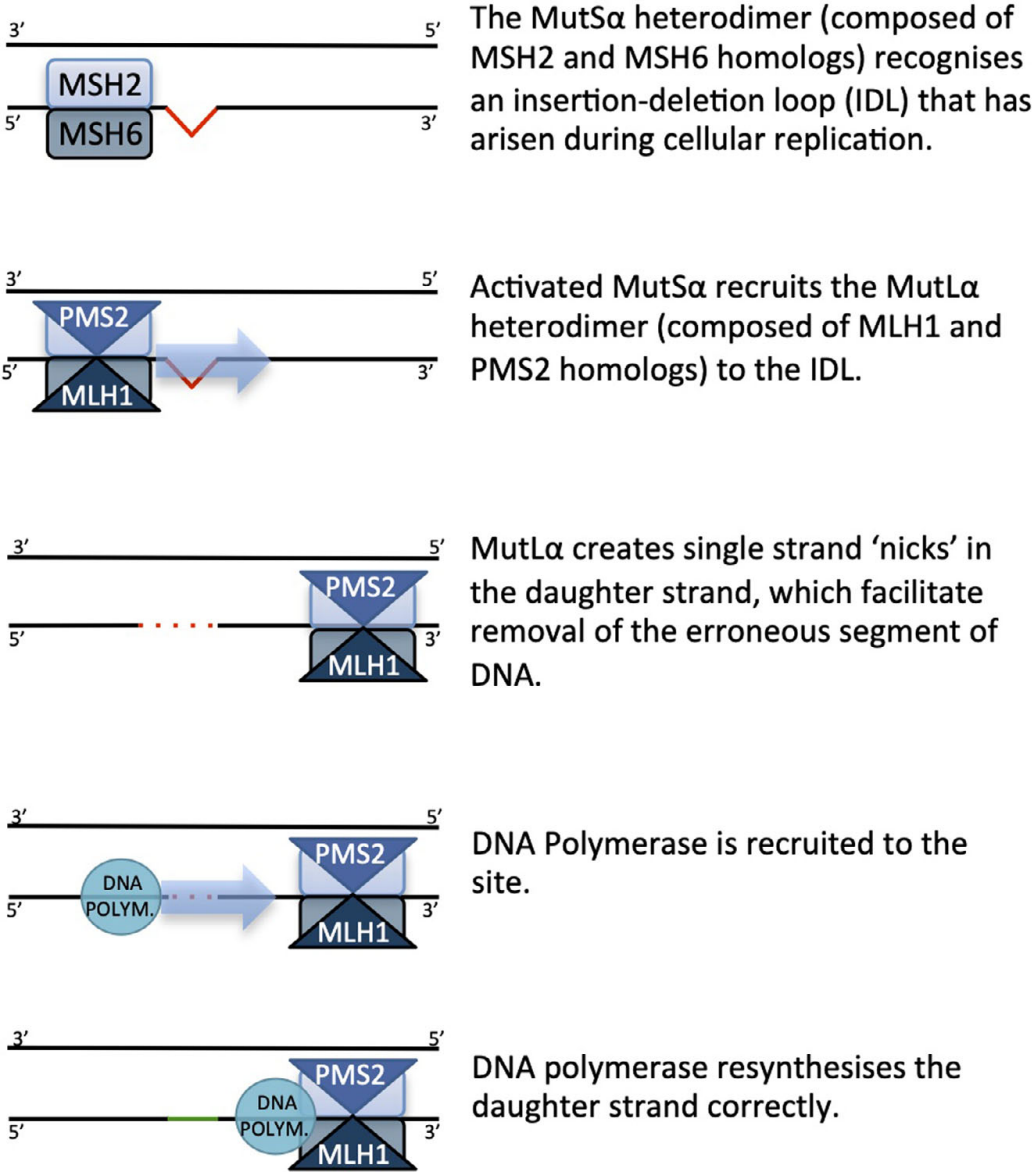
A functional DNA mismatch repair system recognising and removing an insertion/deletion loop that has arisen during cellular replication.

The role of the MMR system in maintaining genomic stability is shown in Figure 2. Without a functioning MMR system, the uncorrected mutation rate accompanying DNA synthesis increases by 1000‐fold.^9^ An individual with Lynch syndrome inherits one pathogenic allele of an MMR gene. In keeping with the Knudson hypothesis, once the second allele acquires a somatic inactivating mutation, the MMR system is nonfunctional, leading to widespread genomic instability as errors made during replication go uncorrected. Hypermutation may eventually lead to carcinogenesis – although it is important to note that in the lifetime of a Lynch syndrome carrier, thousands of cells become MMR‐deficient, but very few cause cancer. This is in part associated with the immune response they elicit. This phenomenon has been observed in the endometrium, where normal glands demonstrate MMR deficiency.^10^

## The epidemiology of Lynch syndrome

The exact prevalence of Lynch syndrome in the general population is unclear. The American Gastroenterological Association estimates the prevalence to be 1 in 440.^11^ A recent study from Denmark estimated the prevalence to be as high as 1 in 278 in the general population.^2^ This would make Lynch syndrome the most common inherited cancer predisposition syndrome. Most (up to 95%) individuals who carry a Lynch syndrome‐causative pathogenic variant are unaware of it.^2^

In cancer populations, a recent systematic review and meta‐analysis concluded that around 3% of endometrial cancers are caused by Lynch syndrome, although the quality of the evidence is poor.^12^ This is equivalent to the rate of Lynch syndrome seen in colorectal cancer,^13^ and current UK guidance from the National Institute for Health and Care Excellence (NICE) supports the universal screening of individuals with colorectal cancer,^14^ and more recently, those with endometrial cancer,^15^ for Lynch syndrome. The number of Lynch syndrome diagnoses associated with ovarian cancer is less clear.^16^ A single centre study found 21% of non‐serous epithelial ovarian cancer to be MMR deficient;^17^ however, there is currently insufficient high quality evidence to give reliable estimates.

Lynch syndrome is not a uniform disorder. The degree of penetrance, disease spectrum and age of cancer onset vary according to the mutated gene.^4^ For example, the incidence of endometrial cancer in *MSH6* pathogenic variant carriers is as high as that in *MLH1* and *MSH2* pathogenic variant carriers,^4^ and the risk is much higher than in *PMS2* pathogenic variant carriers. However, the risk of colorectal cancer associated with *MSH6* is much lower^4^ (Table 1). This has implications for gynaecological surveillance and risk‐reducing strategies.

**Table 1:** The cumulative risk of endometrial and ovarian cancer in women with Lynch syndrome at 40 and 70 years of age, stratified by mutated gene

Gene	Endometrial cancer	
	Cumulative incidence at 40 years % (95% CI)	Cumulative incidence at 70 years % (95% CI)
*MLH1*	3.1 (0.4–5.8)	42.7 (33.1–52.3)
*MSH2*	1.5 (0.0–4.4)	56.7 (41.8–71.6)
*MSH6*	0	46.2 (27.3–65.0)
*PMS2*	0	26.4 (0.8–51.9)

	Ovarian cancer	
	Cumulative incidence at 40 years % (95% CI)	Cumulative incidence at 70 years % (95% CI)
*MLH1*	2.6 (0.1–5.2)	10.1 (4.8–15.4)
*MSH2*	3.8 (0.0–8.0)	16.9 (5.7–28.0)
*MSH6*	4.2 (0.0–12.3)	13. 1 (0.0–31.2)
*PMS2*	0	0

Abbreviations: CI = confidence interval

## Colorectal cancer and Lynch syndrome

Colorectal cancer is the most common and lethal cancer seen in Lynch syndrome carriers. The risk of developing colorectal cancer depends on the affected gene and the sex of the individual. For those with *MLH1* pathogenic variants, the cumulative lifetime risk of colorectal cancer is 47% (95% confidence interval [CI] 39–54%). For those with *PMS2* pathogenic variants, the risk is 14% (95% CI 3–25%).^4^ Lynch syndrome‐associated colorectal cancer has an earlier age of onset, with a crude median age at diagnosis of 52 years versus 69 years in sporadic disease.^11^ Women have a lower penetrance than men, which means their risk of colorectal cancer is less, yet still considerably higher than the general population.^4^

Biennial colonoscopic colorectal surveillance forms the bedrock of management.^11^ High quality surveillance is associated with a significant reduction in deaths from colorectal cancer in Lynch syndrome carriers.^4^ The pathophysiology of Lynch syndrome‐associated colorectal cancers makes colonoscopic detection difficult because tumours arise from flat adenomas that are hard to detect. These cancers have a propensity for the right side of the colon, rather than the rectum or sigmoid colon.^4^ Furthermore, synchronous and metachronous cancers are common, so more extensive surgery with ileo‐sigmoidal or ileo‐rectal anastomosis is often required or preferred.^6^

For the gynaecologist, this information should help counsel women undergoing Lynch syndrome testing because the main effect of a Lynch syndrome diagnosis is the need for regular colonoscopies. Gynaecologists should aim to coordinate surveillance and surgery with their colorectal colleagues, wherever possible.^18^ For example, risk‐reducing gynaecological surgery could be combined with colorectal surveillance or surgery. It is also important to include colorectal colleagues in any relevant clinical communications; Lynch syndrome increases the risk of cancer at multiple sites and care of affected individuals is necessarily multidisciplinary.

## Risk‐reducing strategies in women with Lynch syndrome

Ideally, women with Lynch syndrome should be seen at around the age of 25 years by an expert gynaecologist to learn about the red flag symptoms of cancer, discuss family planning and explore risk‐reducing strategies.^18^ Raising awareness about red flag symptoms empowers women to seek help appropriately. The lifetime risk of gynaecological cancer is sufficiently high to offer total hysterectomy and bilateral salpingo‐oophorectomy for women with Lynch syndrome who have completed childbearing.^19^ The timing of such surgery is gene‐specific, as shown in Table 2. The survival benefit achieved by risk‐reducing surgery is minimal because Lynch syndrome‐associated endometrial and ovarian cancers have a good prognosis. However, for many women with Lynch syndrome, avoiding a cancer diagnosis and the harms associated with its treatment is sufficient to choose risk‐reducing surgery. Preoperative counselling by both a clinical geneticist and gynaecologist is seen as best practice. The laparoscopic approach is preferred because it leads to a shorter recovery time and improved short‐term quality of life;^20^ however, it can be challenging for women who have previously received surgery and/or radiotherapy for colorectal cancer. To reduce a woman’s exposure to multiple surgeries/anaesthetics, where possible, hysterectomy should be coordinated with other risk‐reducing interventions, such as colonoscopy or colorectal surgery. Hysterectomy and bilateral salpingo‐oophorectomy at 40 years of age has been shown to be a cost‐effective strategy.^21^

**Table 2:** An overview of cancer risk‐reducing strategies for women with Lynch syndrome

Considerations	Hysterectomy (± bilateral salpingo‐oophorectomy)	Aspirin	Lifestyle (smoking cessation, reduce weight, increase exercise, healthy diet)	Hormone‐based therapy
*Target population*	Female LS carriers, family completed	All LS carriers, especially those with a raised BMI	All LS carriers	Females of reproductive age
*Timing*	For path_*MLH1* and path_*MSH2* at 35 years For path_*MSH6* at 40 years For path_*PMS2* at 50 years	From 18 years	Any age	From the age of menarche until natural age of menopause
*Mechanism of action*	Removes organs prone to cancer	Not fully understood	General cancer risk factor reduction	Reduced endometrial proliferation, anti‐inflammatory effect
*Evidence*	Retrospective cohorts	Large international randomised controlled studies	Limited evidence in LS populations mostly drawn from non‐LS population and small retrospective cohort data	Retrospective cohort data
*Contraindications*	Surgical and anaesthetic contraindications, wish for future fertility	Peptic ulcer disease, bleeding disorders/haemophilia, severe cardiac failure, active alcohol abuse	Those with pre‐existing health conditions that would prohibit excessive physical exercise	History of estrogen‐dependent or breast cancer, active arterial thromboembolic disease, undiagnosed vaginal bleeding, thrombophilia disorder, history of venous thromboembolism
*Harms*	Surgical harms such as infection, pain, visceral injury, death, etc. Also risks of early menopause (if BSO) such as vasomotor symptoms, increased risk of cardiovascular disease, osteoporosis	Dyspepsia, haemorrhage (usually minor as young population – trial data would support prescription unless any contraindications)	None	Dysuria, skin reactions, mood alterations
*Unknowns*	Whether two‐stage surgical procedure to remove uterus after childbearing and ovaries after menopause improves outcomes	Optimal dosage	The effectiveness of such strategies in LS‐specific cancer risk	Benefit of intrauterine systems in reducing endometrial cancer risk in LS carriers

Abbreviations: BMI = body mass index; BSO = bilateral salpingo‐oophrectomy; EC = endometrial cancer; LS = Lynch syndrome; OC = ovarian cancer; path_ = pathogenic variant

In premenopausal women, bilateral oophorectomy at the time of risk‐reducing hysterectomy results in surgical menopause, causing vasomotor symptoms, urogenital dryness and atrophy and, often, reduced sexual function, emotional lability and cognitive decline. It also increases the risks of osteoporosis, cardiovascular disease and colorectal cancer.^22^ To mitigate these risks, women should be counselled about the benefits of estrogen replacement therapy (ideally a transdermal application) for quality of life and future health. Estrogen has a protective effect against colorectal cancer and does not appreciably increase breast cancer risk.

Women with Lynch syndrome should be encouraged to explore other ways of addressing their cancer risk (Table 2). The risk factors for endometrial cancer in the general population include age, obesity, type 2 diabetes mellitus, nulliparity, early menarche/late menopause and tamoxifen exposure.^23^ There is limited evidence about how lifestyle affects gynaecological cancer risk in women with Lynch syndrome. The oral contraceptive pill is known to reduce the risk of sporadic endometrial and ovarian cancer,^24^, ^25^ as well as *BRCA1/2*‐associated ovarian cancer,^26^ and the levonorgestrel‐releasing intrauterine system reduces the risk of endometrial cancer in the general population.^27^ While there are no conclusive data to support the use of these interventions in women with Lynch syndrome, the prevailing wisdom is that they probably have a beneficial effect on gynaecological cancer risk.

Taking aspirin has been shown to reduce the risk of all cancer types in Lynch syndrome carriers.^5^ Aspirin appears to reduce endometrial cancer risk in obese women with Lynch syndrome compared with nonobese women.^28^ Lifestyle factors may also affect cancer risk in Lynch syndrome carriers. Smoking, alcohol and increased body mass index increase the risk of colorectal cancer in individuals with Lynch syndrome; however, few studies have specifically explored the effect of lifestyle choices on gynaecological cancer risk.^29^ Despite a lack of robust evidence, it would seem sensible for women with Lynch syndrome to eat a healthy diet, maintain a healthy weight, take regular exercise, avoid smoking cigarettes and either abstain from or reduce alcohol intake to a moderate level.

## Gynaecological surveillance in women with Lynch syndrome

Not all women with Lynch syndrome wish to undergo risk‐reducing gynaecological surgery; indeed, fertility‐sparing options are required for those who wish to pursue motherhood.^30^ Gynaecological surveillance aims to reassure women or detect cancer at a precancerous or early stage to improve morbidity and survival outcomes. Trials have investigated many modalities (Table 3). Transvaginal ultrasound has limited utility for detecting endometrial abnormalities in premenopausal women, as endometrial thickness fluctuates naturally during the menstrual cycle. On the other hand, hysteroscopy and endometrial biopsy are invasive procedures, with 30–40% of women suffering pain during their completion. Overall, data relating to gynaecological surveillance are of low quality, with a predominance of single‐centre, retrospective studies. The results are contradictory, with some studies showing benefit and others not.^18^ Many women diagnosed with gynaecological cancers through surveillance were symptomatic at the time. Furthermore, endometrial cancer survival rates in women with Lynch syndrome are extremely good anyway, with a 10‐year survival of 90% or more.^31^ Thus, the benefit for endometrial cancer‐specific survival is uncertain. The literature does not support gynaecological surveillance for improving outcomes from ovarian cancer in Lynch syndrome. The United Kingdom Familial Ovarian Cancer Screening Study (UKFOCS) found that a combination of serum CA125 and transvaginal ultrasound scanning was sensitive and led to a stage shift in disease in women with a lifetime risk of ovarian cancer >10%. However, few Lynch syndrome‐associated ovarian cancers informed this analysis.^32^

**Table 3:** Gynaecological surveillance methodologies currently used in women with Lynch syndrome

Type of cancer	Surveillance method	Benefit	Disadvantage	Estimated sensitivity (%)	Estimated specificity (%)
Endometrial cancer	Pelvic ultrasound	Cheap, widely accessible, acceptable to women, minimal complications, can assess ovaries	In premenopausal women, difficult to interpret; no tissue diagnosis; risk of incidental findings	15–100	55–100
	Endometrial biopsy	Outpatient procedure, tissue diagnosis, widely accessible	Painful, risk of infection/perforation, sampling error, need for repeat procedure	80–100	60–100
	Outpatient hysteroscopy ± directed biopsy	Outpatient procedure, tissue diagnosis, widely accessible, target biopsy	Small evidence base in LS, risk of infection/perforation, visceral injury, relatively expensive, can be prohibitively painful	90–100	90–100
Ovarian cancer	Pelvic ultrasound	Cheap, widely accessible, acceptable to women, minimal complications, can assess endometrium	Small evidence base in LS, high rate of incidental findings leading to unnecessary interventions	10–60	40–100
	Serum CA125	Cheap, widely accessible, acceptable to women, minimal complications, can be done in primary care	Small evidence base in LS, nonspecific and therefore can lead to unnecessary anxiety and intervention	20–58	80–98
	Combined (CA125 + pelvic ultrasound)	Cheap, widely accessible, acceptable to women, minimal complications, can assess endometrium, improved sensitivity compared with ultrasound alone	As above	70–89	80–99

NB: Sensitivity and specificity data for ovarian cancer is taken from wild type and other high‐risk populations; the figures in women with Lynch syndrome are not known. CA125 = cancer antigen 125; LS = Lynch syndrome

## Fertility and Lynch syndrome

There is no evidence that Lynch syndrome has any effect on fertility. However, as an autosomal dominant condition, carriers of Lynch syndrome have a 50% chance of passing on the defective MMR gene to their children. Lynch syndrome is on the list of conditions curated by the Human Fertilisation and Embryology Authority (HFEA), for which those affected can access pre‐implantation genetic testing (PGT). PGT allows embryos lacking the MMR pathogenic variant to be selected for transfer following in vitro fertilisation (IVF). This reduces the risk of transmission, but IVF is a demanding process and many couples affected by Lynch syndrome prefer to conceive naturally, whatever the risk. The uptake of PGT by women or their partners affected by Lynch syndrome is variable, but patient survey data indicate a significant minority would consider it.^33^ In the UK, it is convention for genetic counsellors to lead on referral for PGT; however, gynaecologists may be asked for advice, so they should know what is possible and what is involved. Those wishing to conceive naturally should be advised that the risk of endometrial cancer rises sharply for women older than 40 years and may frustrate pregnancy plans that are left too late.

## Screening gynaecological cancers for Lynch syndrome

The prevalence of Lynch syndrome in women with endometrial and ovarian cancer is around 3% and 1–2%, respectively.^12^, ^34^ There is an emerging consensus that all women with endometrial cancer should be screened for Lynch syndrome, where resources permit.^18^ Indeed, this is what NICE recommends.^15^ Where resources are limited, testing can be restricted to those who develop endometrial cancer under the age of 70 years, or where other clinical features are suggestive of Lynch syndrome; for example, a strong family history of Lynch syndrome‐associated cancers.^18^

## Diagnosing Lynch syndrome in women with endometrial cancer

### Clinical criteria

Warthin and Lynch discovered Lynch syndrome through careful documentation of their patients’ pedigrees. The importance of taking a detailed family history in an oncology clinic cannot be overestimated. The Amsterdam II criteria^35^ and revised Bethesda guidelines^36^ are age and family history‐based prediction tools that were designed to target Lynch syndrome testing in colorectal cancer. Use of these tools in endometrial cancer has been explored in several studies, and the reported specificity is 61% and 49% for Amsterdam II criteria and revised Bethesda guidelines, respectively.^37^ Unfortunately, such family history scores have very low sensitivity to identify *MSH6* or *PMS2* pathogenic variant carriers.^38^ The newer prediction tools MMRpredict,^39^ MMRpro^40^ and PREMM_5_ ^41^ have increased diagnostic accuracy. MMRpredict has a reported sensitivity of 94% and a specificity of 91% for *MLH1* and *MSH2* pathogenic variant carriers, while discrimination of *MSH6* was more difficult and *PMS2* was not assessed.^42^ A head‐to‐head comparison of these new family history‐based tools concluded that MMRpro and PREM_1,2,6_ could be implemented in both clinical and population settings using a risk cut‐off of 5%.^43^ However, the precision of these tools relies on the patient describing, and the clinician recording, an accurate family history. This is not always practical in busy outpatient departments. If your patient has a particularly strong family history of cancer, it is best to seek advice from your local clinical genetics service.

Women with Lynch syndrome develop endometrial cancer at an earlier age than those with sporadic tumours.^18^ While younger women may be more likely to have Lynch syndrome‐associated endometrial cancer, restricting Lynch syndrome testing to women under the age of 50 years would miss cases of Lynch syndrome. The same is true for histological subtype; endometrioid endometrial and ovarian tumours^34^ are most commonly associated with Lynch syndrome, but other histological subtypes have been reported.^44^ It is widely held that restricting Lynch syndrome testing according to clinical parameters is imperfect and that tumour‐based testing is the most effective way of triaging women for germline analysis.^45^

### Tumour‐based testing

**Figure 3:**
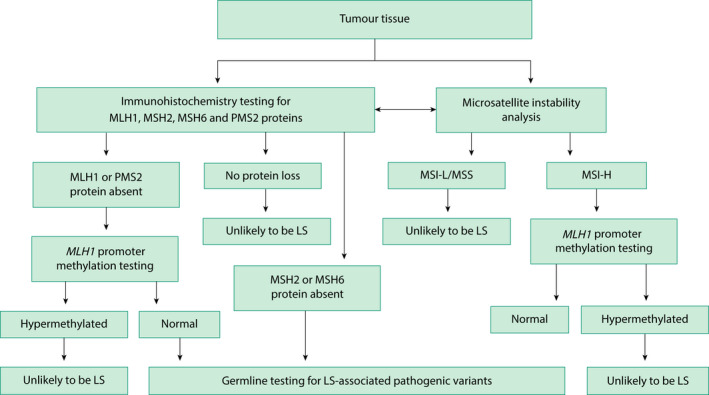
Outline of a potential diagnostic schema used to risk stratify tumours for Lynch syndrome. Abbreviations: IHC = immunohistochemistry; LS = Lynch syndrome; MMR = mismatch repair; MSI‐H = microsatellite instability high; MSI‐L = microsatellite instability low; MSS = microsatellite stable. *Indicates that IHC and MSI‐based tumour triage can be used in combination or individually.

A defective MMR system leads to phenotypical features within the tumour. When a pathogenic variant is acquired within a gene, it affects the expression of that gene’s corresponding protein, either through the amount of protein produced or changes in its structure and function. Tumour‐based testing does not identify people with Lynch syndrome; it stratifies their risk for the condition. This is important because it is widely accepted that tumour‐based tests can be done without explicit consent.^18^ They are used to identify individuals who should undergo definitive, but expensive, germline testing to ensure testing strategies remain cost effective^46^ (Figure 3).

#### Immunohistochemistry

**Figure 4:**
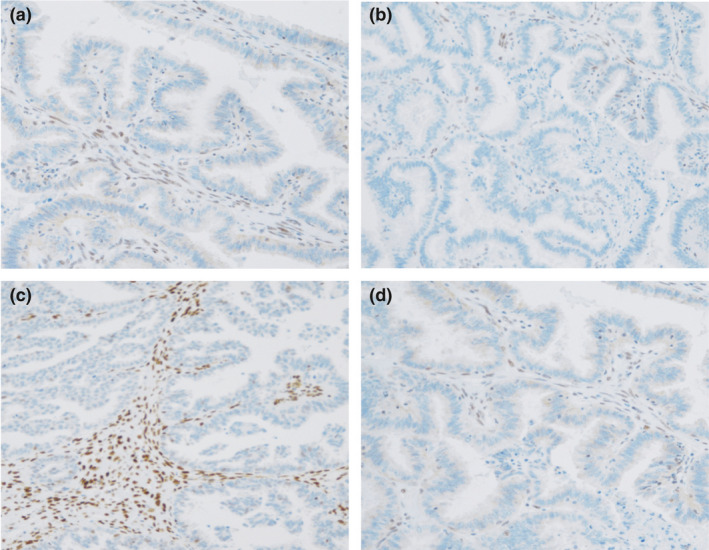
Mismatch repair immunohistochemistry showing loss of A: MLH1, B: MSH2, C: MSH6 and D: PMS2 protein in endometrial cancer glands, with conserved expression in stromal tissue.

Loss of tumour expression of one or more MMR proteins, known as MMR deficiency, is a feature of Lynch syndrome (Figure 4). MMR protein immunohistochemistry has a sensitivity of 80–100% and a specificity of 60–80% for detecting Lynch syndrome‐associated endometrial cancer.^18^ The relative lack of specificity is associated with somatic loss of MMR expression – usually as a consequence of hypermethylation of the promoter region of the *MLH1* gene.^47^, ^48^ *MLH1* methylation testing correctly identifies tumours caused by somatic methylation events, thereby reducing the proportion of patients who need to undergo definitive germline Lynch syndrome testing.

#### Microsatellite instability testing

Microsatellites are small DNA motifs that are repeated throughout the genome.^49^ They comprise 2–5 nucleotides repeated 5–50 times. These sequences are highly conserved in the offspring of an individual; however, there is marked variation across a population.^50^ Microsatellite instability (MSI) is a marker of hypermutation as seen in Lynch syndrome‐associated tumours.^51^ As Lynch syndrome tumours have multiple insertion/deletion mutations, there is inevitably variation within the tumour microsatellites. Detecting these variations gives a means of diagnosis through polymerase chain reaction (PCR) testing. Tumours are categorised as MSI‐stable (MSS), or MSI‐low (MSI‐L) if <30% of markers are unstable, and MSI‐high (MSI‐H) if >30% of markers are unstable; this is the category to which most Lynch syndrome tumours belong. Sporadic tumours can also be MSI‐H;^40^ usually as a result of hypermethylation of the promoter region of *MLH1*. The diagnostic accuracy of MSI testing has been reported as high, with a sensitivity of 92% and a specificity of 59% in colorectal cancer, where most research has concentrated.^52^ Similar accuracy is reported for endometrial cancer, although the number of tested tumours and the quality of available studies is much lower.^18^ There is good reported concordance between MSI and immunohistochemistry testing for Lynch syndrome tumour identification,^18^ but more recent data suggest that MSI testing is less accurate in endometrial cancer – particularly at identifying *MSH6* carriers.^53^

#### Genomic diagnosis

Genomic testing of the tumour or the patient is referred to as somatic and germline testing, respectively. Both are done using next‐generation sequencing (NGS). While germline testing is the only means by which a diagnosis of Lynch syndrome can be made, it is not always straightforward. First, the *PMS2* gene is very hard to sequence, so it can only be done in specialist centres. Second – and more importantly – when a gene is sequenced, a list of bound nucleotides (A, C, T, G) is generated; an error in this list does not always have a pathological consequence. Sequencing is analogous to detecting spelling errors in a book: the meaning of those spelling errors is sometimes very hard to deduce. If you spell the word ‘cosy’ or ‘cozy’, it has the same meaning. If, however, you change ‘now’ to ‘not,’ the meaning is very different. When the meaning of a mutation/pathogenic variation cannot be determined it is classified as a variant of unknown significance (VUS). The determination and management of individuals with VUS is best left to geneticists. Germline sequencing is the definitive test for Lynch syndrome and must always be preceded by informed consent taken by a trained individual.

## Targeted treatments in Lynch syndrome‐associated gynaecological cancers

**Figure 5:**
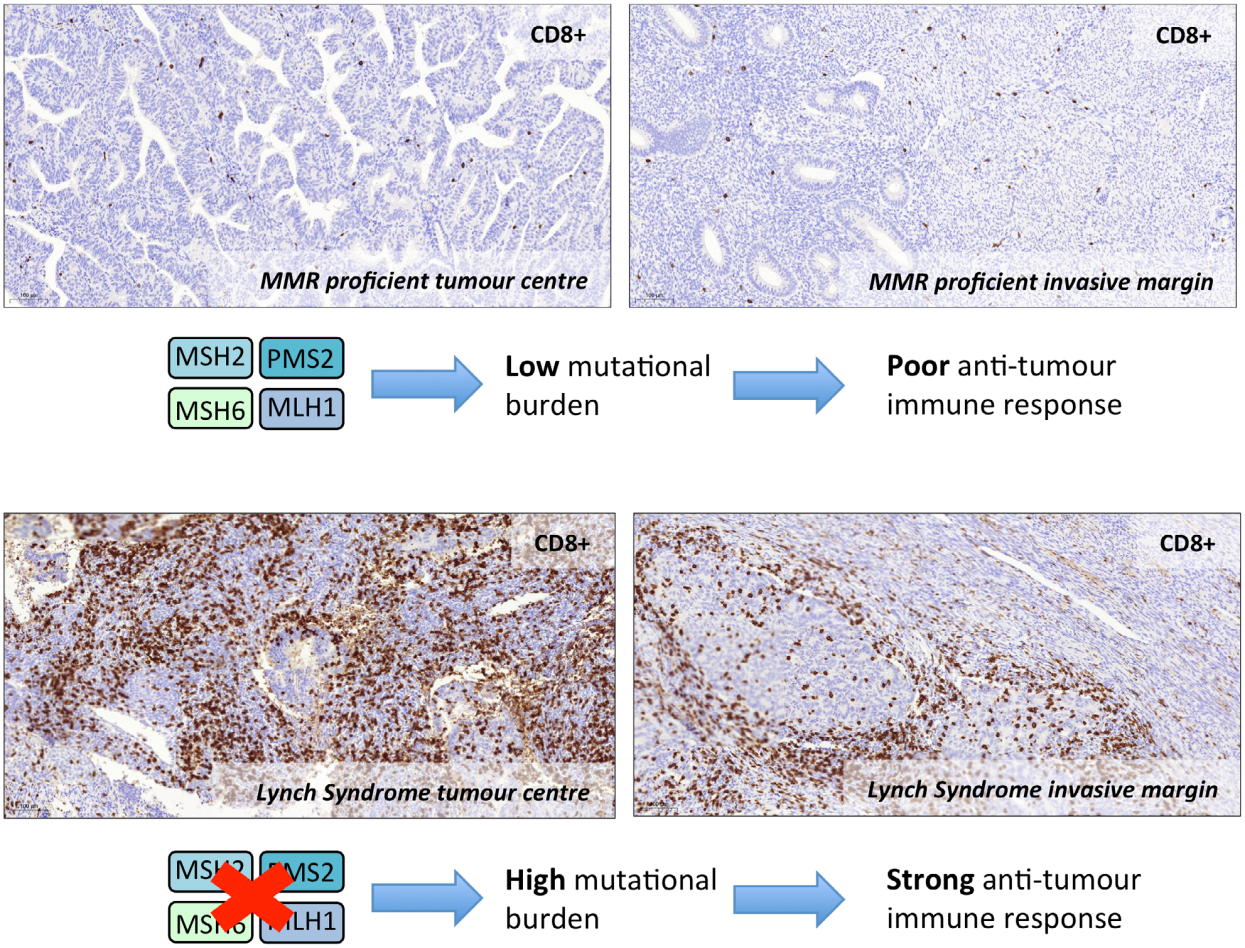
Comparison of CD8+ cytotoxic T‐cell infiltration in a sporadic mismatch repair proficient endometrial cancer (top panels) with a Lynch syndrome‐associated endometrial cancer (bottom panels). The abundance of neoantigens expressed by Lynch syndrome‐associated tumours leads to enhanced immunogenicity and a robust anti‐cancer T‐cell response.

MMR‐deficient cancers have certain characteristics that are important when planning treatment and follow‐up. These tumours are very immunogenic, eliciting a marked and unique immune response (Figure 5).^54^ The main mechanism of immune evasion seen in MMR‐deficient cancers is exploitation of the PD‐1/PD‐L1 pathway.^55^ This is a druggable pathway, which has been explored in recent clinical trials with excellent results.^56^ The PD‐1 checkpoint inhibitor pembrolizumab is an IgG4 isotype antibody that targets the PD‐1 receptor expressed by peripheral lymphocytes. It binds and blocks the PD‐1 receptor, preventing its activation by the cancer.^57^ It is one of few drugs to be licenced by the United States Food and Drug Administration for all tumours of a specific phenotype; in this case, those that are MSI‐H or MMR‐deficient, as opposed to those originating at a particular site.^58^ Lynch syndrome‐associated gynaecological cancers have improved survival outcomes compared with sporadic cancers.^34^, ^59^ This is important when counselling patients regarding prognosis. It may also enable shorter or less intensive follow‐up; however, more data are needed before definitive recommendations can be made.

## The future

Many unknowns remain regarding Lynch syndrome and its associated gynaecological cancers. The benefits and harms of gynaecological surveillance and the effectiveness of risk‐reducing interventions, particularly oral and intrauterine progestins, have yet to be established. Novel strategies are being tested to harness the Lynch syndrome patient’s own immune system to prevent cancers through vaccination.^60^ Novel diagnostic methods, with the potential for complete automation, are in development. Such technologies would simplify and reduce the costs of Lynch syndrome screening and diagnostic pathways.

## Key resources

One published guideline, written by the Manchester International Consensus Group, looks specifically at the gynaecological manifestations of Lynch syndrome and offers clear and comprehensive guidance for clinicians and patients.^18^ The European Hereditary Tumour Group^61^ produces broad guidelines on the clinical management of Lynch syndrome, with guidance reviewed and updated regularly. The prospective Lynch syndrome database^62^ has produced a risk prediction tool that clinicians can use to identify an individual patient’s risk of developing cancer as they age, enabling more personalised management. For patient support and information, Lynch Syndrome UK (LSUK)^63^ is a patient support group with excellent resources. Finally, the PREMM_5_ model^64^ is useful for directing family history‐taking during initial consultations with patients. High scores (>5%) should prompt referral to the local clinical genetics team. All those with a score >2.5% should have tumour testing (if applicable) for Lynch syndrome, according to the algorithm.

### Disclosure of interests

There are no conflicts of interest.

### Contribution to authorship

EJC is this article’s guarantor. NAJR and EJC designed and wrote the article. NCR aided in design of the figures and tables. RFTM, MWS and DGE provided expert material and review. All authors provided critical comment, edited the manuscript, and approved its final version.

## Funding

NAJR is a Doctoral Medical Research Council (MRC) Research Fellow (MR/M018431/1). DGE is a National Institute for Health Research (NIHR) Senior Investigator (NF‐SI‐0513‐10076). DGE and EJC are supported through the NIHR Manchester Biomedical Research Centre (IS‐BRC‐1215‐20007).

## Supporting information


**Infographic S1.** Lynch syndrome for the gynaecologist
