# Supplementary material for: Lynch syndrome for the gynaecologist
Source: Obstet Gynaecol. 2021 Jan 18;23(1):9–20. doi: 10.1111/tog.12706 (PMC7898635; doi:10.1111/tog.12706)
Supplement: Supplementary file 1 — Infographic S1. Lynch syndrome for the gynaecologist [file TOG-23-9-s001.pdf]

Lynch syndrome
for the gynaecologist

Lynch syndrome is an inherited predisposition to several cancer types, including endometrial, colorectal and ovarian cancer. The gynaecologist has a crucial role in diagnosing Lynch syndrome and advising women of its implications.

With the emerging integration of genomic medicine into routine clinical practice, obstetricians and gynaecologists must become more familiar with common genetic conditions, such as Lynch syndrome

What is Lynch syndrome?

Lynch syndrome is an autosomal dominant cancer predisposition syndrome arising from a dysfunctional DNA mismatch repair (MMR) system

Up to 95% of Lynch syndrome carriers are unaware

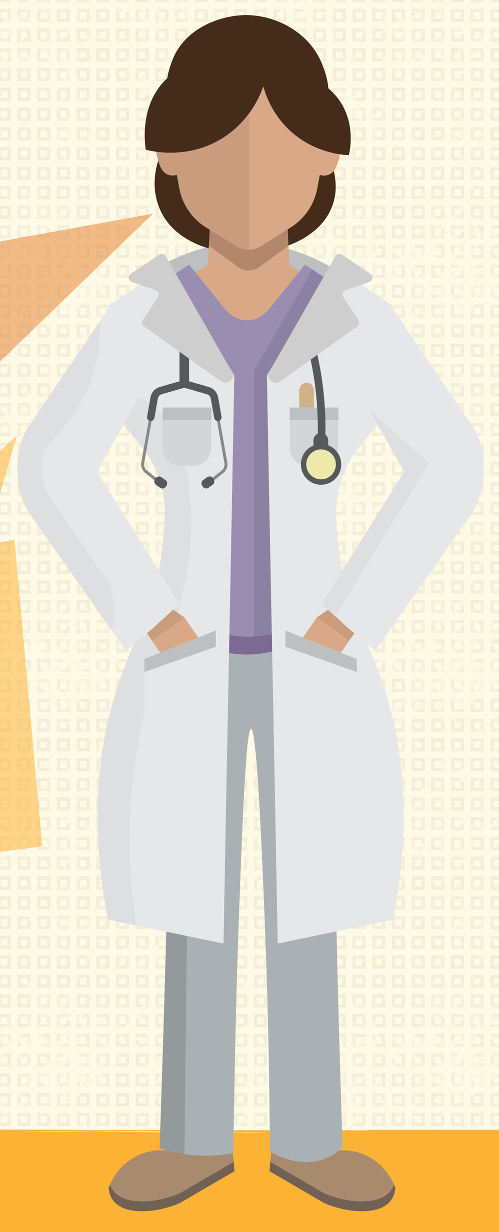

Lynch syndrome may occur in up to

1 in 278 people

making it the most common inherited cause of cancer

Lynch syndrome arises from inherited mutations, known as pathogenic variants, affecting *MLH1*, *MSH2*, *MSH6* and *PMS2*, the MMR genes responsible for ensuring fidelity during DNA replication

Cumulative lifetime risks of cancer in Lynch syndrome

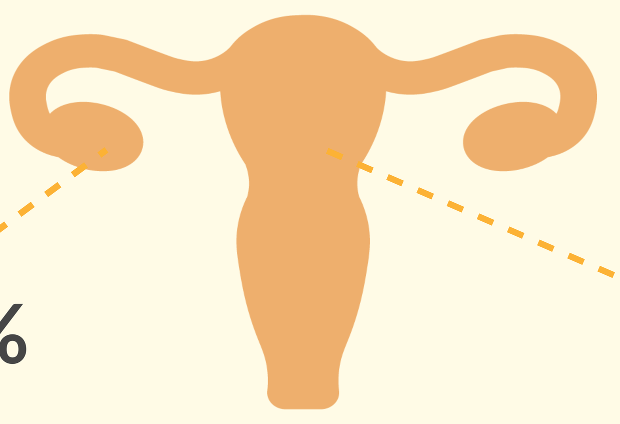

Ovarian
Up to 17%

Endometrial
Up to 57%

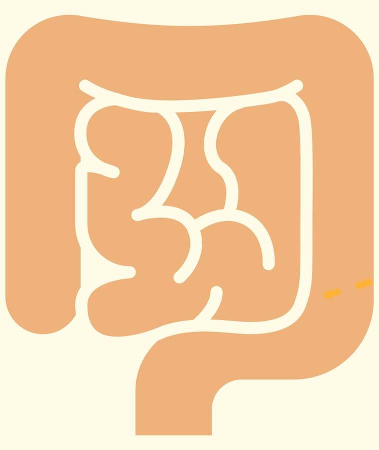

Colorectal
Up to 47%

Women with Lynch syndrome should be seen at around the age of 25 by an expert gynaecologist to learn about the red flag symptoms of cancer, discuss family planning and explore cancer risk-reducing strategies

Screening and diagnosis

The National Institute for Health and Care Excellence now recommends that all women with endometrial cancer are screened for Lynch syndrome

Tumour-based testing

Tumour-based testing does not identify people with Lynch syndrome; it stratifies their risk for the condition.

Immunohistochemistry

Immunohistochemistry tests for loss of MMR protein expression (MMR deficiency). There is a relative lack of specificity, associated with somatic loss of MMR expression.

Microsatellite instability analysis

Microsatellites are repeated DNA motifs. Instability is a marker of hypermutation, as seen in Lynch syndrome-associated tumours. If microsatellite instability is high, Lynch syndrome is more likely.

Germline testing

Involving genomic testing of the patient, germline testing is the only way in which a Lynch syndrome diagnosis can be made. It is done using next-generation sequencing, is expensive and can only be done in specialist centres.

Risk-reducing strategies

Hysterectomy

The lifetime risk of gynaecological cancer is sufficiently high to offer total hysterectomy +/- bilateral salpingo-oophorectomy for women with Lynch syndrome who have completed childbearing.

Hormone therapy

The oral contraceptive pill reduces the risk of sporadic ovarian and endometrial cancer, and the levonorgestrel-releasing intrauterine system reduces the risk of endometrial cancer in the general population, so it is thought these may also reduce cancer risk in Lynch syndrome.

Aspirin

Aspirin has been shown to reduce the risk of cancer in Lynch syndrome. Trials to determine the best dose of aspirin for cancer prevention are ongoing.

Lifestyle modifications

While few studies have specifically explored the effect of lifestyle choices on cancer risk in Lynch syndrome, smoking cessation, maintaining a healthy body mass index and increased exercise are thought sensible.

Gynaecological surveillance

There is currently no strong evidence to support gynaecological surveillance for the early detection of gynaecological cancer in Lynch syndrome.

The future...

Novel strategies are being tested to harness the Lynch syndrome patient's own immune system to prevent cancers through vaccination. Novel diagnostic methods, with the potential for complete automation, are in development; such technologies would simplify and reduce the costs of Lynch syndrome screening and diagnostic pathways.

This is a summary of a review published in TOG. For further details on Lynch syndrome, please read the full article:
Ryan NAJ, McMahon RFT, Ramchander NC, Seif MW, Evans DG, Crosbie EJ. Lynch syndrome for the gynaecologist. The Obstetrician & Gynaecologist 2021; https://doi.org/10.1111/tog.12706

onlinetog.org
